# Inflammatory activity stratification improves liver stiffness diagnosis of fibrosis in autoimmune hepatitis

**DOI:** 10.3389/fimmu.2026.1865212

**Published:** 2026-06-10

**Authors:** Yan Huang, Delin Liu, Yunjiang Li, Xu Zhang, Yu Tang

**Affiliations:** 1 Hangzhou Xixi Hospital, Affiliated to Zhejiang Chinese Medical University (Hangzhou Sixth People’s Hospital), Hangzhou, China; 2 The Second Affiliated Hospital of Jiaxing University, Jiaxing, China

**Keywords:** autoimmune hepatitis, correction model, elastography, hepatic fibrosis, inflammation stratification, liver stiffness measurement

## Abstract

**Objective:**

To investigate the correction value of inflammatory activity stratification on the diagnostic performance of liver stiffness measurement (LSM) for hepatic fibrosis in patients with autoimmune hepatitis (AIH), and to provide evidence for accurate fibrosis assessment in AIH patients.

**Methods:**

Clinical data of AIH patients who underwent liver biopsy at our hospital from January 2017 to January 2026 were retrospectively analyzed. All patients underwent LSM by transient elastography within the same period. Patients were divided into the G1–G2 group (mild-to-moderate inflammation) and G3–G4 group (severe inflammation) according to the simplified Scheuer inflammatory grade. Liver pathological fibrosis staging (S0–S4) served as the gold standard. Receiver operating characteristic (ROC) curve analysis was used to evaluate the diagnostic performance of LSM. A correction model was constructed by multivariate logistic regression and internally validated using the Bootstrap method with 1000 resamplings.

**Results:**

A total of 86 AIH patients were finally enrolled, including 79 females (91.9%) with a mean age of (54.1 ± 9.1) years. There were 52 patients in the G1–G2 group and 34 in the G3–G4 group. In the overall cohort, the area under the ROC curve (AUC) of LSM for diagnosing S≥2 fibrosis was 0.73 (95% CI: 0.62–0.83), with an optimal cutoff of 8.2 kPa. After inflammation stratification, the AUC in the G1–G2 group (0.85, 95% CI: 0.76–0.94) was significantly higher than that in the G3–G4 group (0.69, 95% CI: 0.54–0.84) (Z = 2.13, P = 0.033). The optimal cutoffs were 7.8 kPa and 9.1 kPa, respectively. A correction formula was established for the G3–G4 group: corrected LSM = raw LSM − 0.4 × inflammatory grade. After correction, the AUC for S≥2 increased to 0.85 (95% CI: 0.76–0.93), sensitivity from 55.0% to 86.7%, and specificity from 88.5% to 69.2% (all P<0.05).

**Conclusion:**

The diagnostic performance of LSM for fibrosis in AIH patients is affected by inflammatory activity, and severe inflammation leads to pseudo-elevation of LSM. The inflammation-stratified correction model significantly improves diagnostic accuracy and optimizes fibrosis staging in AIH patients. Further validation is required prior to clinical implementation.

## Introduction

Autoimmune hepatitis (AIH) is an autoimmune disease mediated by autoantibodies and T cells directed against hepatocytes, characterized by chronic progressive hepatic parenchymal inflammation and fibrosis. Without timely intervention, the 5−year incidence of cirrhosis can reach 40% (1). Therefore, early and accurate assessment of hepatic fibrosis is a core component in the management of AIH. Patients with mild fibrosis (S0–S1) can be managed with immunosuppressants to control inflammatory progression, whereas those with moderate or advanced fibrosis (S≥2) require intensified treatment and close prognostic monitoring to prevent progression to cirrhosis (2).

Liver biopsy is the gold standard for diagnosing hepatic fibrosis. However, as an invasive procedure, it carries risks of complications such as bleeding and infection. Moreover, approximately 15% of patients cannot receive accurate staging due to sampling error (3, 4). Commonly used serological fibrosis markers, including the fibrosis index based on the four factors (FIB−4), aspartate aminotransferase to platelet ratio index (APRI), and hyaluronic acid, are non−invasive but show limited diagnostic performance in AIH patients (5).

AIH is characterized by immune-mediated persistent inflammation, and serological markers are easily affected by fluctuations in inflammatory activity, such as aspartate aminotransferase (AST) and alanine aminotransferase (ALT). These markers cannot distinguish elevation caused by inflammation from that caused by fibrosis. Multiple studies have confirmed that the AUC for diagnosing S≥2 fibrosis is mostly below 0.70, limiting their clinical utility and failing to meet the demand for precise fibrosis evaluation in AIH (6, 7).

In recent years, ultrasound elastography techniques, including transient elastography (TE) and two−dimensional shear wave elastography (2D−SWE), have been widely used for non−invasive fibrosis assessment by measuring liver stiffness measurement (LSM) in viral hepatitis and non−alcoholic fatty liver disease, with diagnostic performance recommended by multiple guidelines (8). However, the pathological features of AIH differ significantly from those of other liver diseases. AIH typically exhibits portal and interface hepatitis with prominent plasma cell infiltration and highly variable inflammatory activity (9).

LSM is influenced not only by fibrosis but also by hepatocellular swelling and sinusoidal congestion secondary to inflammation, leading to pseudo-elevation of liver stiffness. Such inflammation−related interference may cause misinterpretation of LSM in AIH, such as misclassifying stiffness elevation due to severe inflammation as advanced fibrosis, leading to overtreatment (10).

Current studies on LSM for fibrosis in AIH have two major limitations. First, most studies are small-sample or include mixed liver disease populations, lacking dedicated research on pure AIH cohorts that exclude confounding effects of other liver diseases (11). Second, few studies account for stratification by inflammatory activity, reporting only a single diagnostic cutoff for the overall population and failing to address the clinical challenge of variable interpretation under different inflammatory states. Globally, only 2 published studies on LSM in AIH mention inflammation but do not establish a targeted correction strategy (12).

Accordingly, this study systematically evaluated the diagnostic performance of LSM for significant hepatic fibrosis (S≥2) in AIH patients across different grades of inflammatory activity and developed a practical LSM correction model for patients with severe inflammation (G3–G4). We aim to provide a dedicated non−invasive fibrosis assessment scheme for AIH, reduce unnecessary liver biopsies, and optimize clinical management.

## Patients and methods

### Study population

This retrospective study was approved by the Ethics Committee of Hangzhou Xixi Hospital (approval No. 2021−46). All clinical data, pathological results, and ultrasound images were fully anonymized before analysis by removing all patient identifiers to ensure strict compliance with privacy regulations. Given the retrospective and non−interventional nature of this study, the Ethics Committee formally waived the requirements for written informed consent in accordance with the Declaration of Helsinki.

This study was designed and reported in accordance with the STROBE statement for observational cohort studies and the TRIPOD guideline for multivariable prediction model development and validation. AIH patients who underwent liver biopsy at our hospital between January 2017 and January 2026 were retrospectively enrolled.

### Inclusion criteria

Met the diagnostic criteria of the Guidelines for the Diagnosis and Treatment of Autoimmune Hepatitis (2021 Edition) (13), including elevated serum ALT, positive autoantibodies (ANA, SMA, etc.), and liver histology consistent with AIH (portal and interface hepatitis with plasma cell infiltration); Underwent LSM within 1 week before liver biopsy; Had complete clinical data, including age, sex, body mass index (BMI), liver function, and history of immunosuppressive therapy.

### Exclusion criteria

Complicated with viral hepatitis, non−alcoholic fatty liver disease, primary biliary cholangitis, or other liver diseases; Inadequate liver biopsy specimen (≤5 portal tracts on microscopy); Received glucocorticoid pulse therapy within 1 month before LSM; Presented with cirrhotic complications (ascites, esophagogastric varices) or severe fatty liver.

## Instruments and methods

### Liver stiffness measurement

Liver stiffness (kPa) and controlled attenuation parameter (dB/m) were measured using a FibroTouch (14) liver fibrosis analyzer (HiSky, China) equipped with a dynamic broadband probe (2.5 MHz). All examinations were performed by a senior sonographer with more than 5 years of experience in liver elastography to minimize inter−operator variability.

The right liver lobe was selected, avoiding anatomical structures such as the gallbladder, large vessels, and superficial subcutaneous fat. Patients fasted for at least 8 hours and lay supine with the right arm raised to fully expose the right intercostal space. Scanning was performed in the 7th to 9th intercostal spaces between the anterior axillary line and midaxillary line.

A region of liver parenchyma 1.5–2.0 cm beneath the liver capsule, homogeneous in texture, and free of large vessels and bile ducts was chosen as the sampling area. Patients were instructed to hold their breath briefly after quiet respiration. The transient elastography mode was activated, and images were frozen when stable and uniformly filled.

A region of interest (ROI) was manually placed within the sampling frame, and the system automatically calculated and displayed the mean LSM (kPa). Each patient underwent 10 repeated measurements at the same site, and the median value was used as the final LSM. A 60% success rate and a coefficient of variation <15% among 10 measurements were required for valid results.

### Liver histopathology

Liver tissue specimens were obtained using an 18G needle under ultrasound guidance. Specimens were required to be ≥15 mm in length and contain at least 6 complete portal tracts on microscopy. Two senior pathologists with more than 10 years of experience in liver pathology independently reviewed slides in a blinded manner.

Specimens were fixed in 4% formaldehyde, embedded in paraffin, and stained with hematoxylin and eosin (HE) and Masson trichrome. Inflammatory activity grade (G1–G4) and fibrosis stage (S0–S4) were assessed according to the simplified Scheuer scoring system. Discrepancies were resolved by consensus review. Inter−observer agreement was evaluated using Kappa statistics: Kappa = 0.88 for inflammatory grade, 0.90 for fibrosis stage, and 0.89 overall (P <0.001), indicating excellent agreement.

### Outcome measures

Baseline characteristics: age, sex, BMI, history of AIH treatment (untreated/immunosuppressive therapy ≥6 months). LSM values, Pathological indices: inflammatory grade (G), fibrosis stage (S). Primary endpoint: significant fibrosis (S≥2); Secondary endpoint: advanced fibrosis (S≥3).

### Statistical analysis

#### Sample size calculation

Based on previous meta-analyses, the AUC of LSM for diagnosing significant fibrosis (S≥2) in autoimmune hepatitis (AIH) was reported to be approximately 0.80 in unstratified cohorts (11). However, these estimates did not account for the confounding effect of inflammatory activity, which may lead to biased interpretation in clinical practice. Sample size calculation was performed using PASS 15.0 with a type I error rate (α) of 0.05, statistical power (1−β) of 0.80, and null hypothesis AUC of 0.50. The minimum required sample size was estimated to be 55 patients. The present study enrolled 86 patients, providing sufficient statistical power to evaluate the stratified and corrected performance of LSM. However, the initial calculation was based on the overall cohort. For the primary comparison in the G3–G4 subgroup (raw LSM vs. corrected LSM), *post-hoc* power analysis was performed and confirmed adequate statistical power.

Statistical analyses were performed using SPSS 26.0 (IBM Corp., Armonk, NY, USA) and R 4.2 (R Foundation for Statistical Computing, Vienna, Austria). Graphs were plotted using GraphPad Prism 9.0 (GraphPad Software, San Diego, CA, USA). Continuous data were expressed as mean ± standard deviation (x ± s). Between−group comparisons used independent−samples t−test (normal distribution) or Mann−Whitney U test (non−normal distribution). Categorical data were expressed as number (percentage). Between−group comparisons used χ² test or Fisher exact test (expected frequency <5). Inter−observer agreement for pathological evaluation was assessed using Kappa test; Kappa >0.85 indicated excellent agreement. The sensitivity and specificity before and after correction were compared using the McNemar’s exact test.

Receiver operating characteristic (ROC) curve analysis was used to evaluate the diagnostic performance of LSM for significant fibrosis (S≥2) and advanced fibrosis (S≥3). The area under the curve (AUC), optimal cutoff, sensitivity, specificity, positive predictive value (PPV), and negative predictive value (NPV) were calculated. Pairwise comparisons of AUCs were performed using the DeLong test. *Post-hoc* power analysis was conducted for the G3–G4 subgroup (n=34) to verify the adequacy of sample size for comparing the AUC of raw LSM and corrected LSM using the DeLong test, with a two-sided α=0.05.

Multivariate logistic regression was used to construct a correction model with significant fibrosis (S≥2) as the dependent variable and raw LSM, inflammatory grade, and LSM × inflammatory grade interaction as independent variables. Regression coefficients (β), odds ratios (OR), and 95% confidence intervals (CI) were recorded. Internal validation was performed using the Bootstrap method with 1000 resamples to assess model stability. A two−sided P<0.05 was considered statistically significant.

## Results

### Patient baseline data

**Figure 1:**
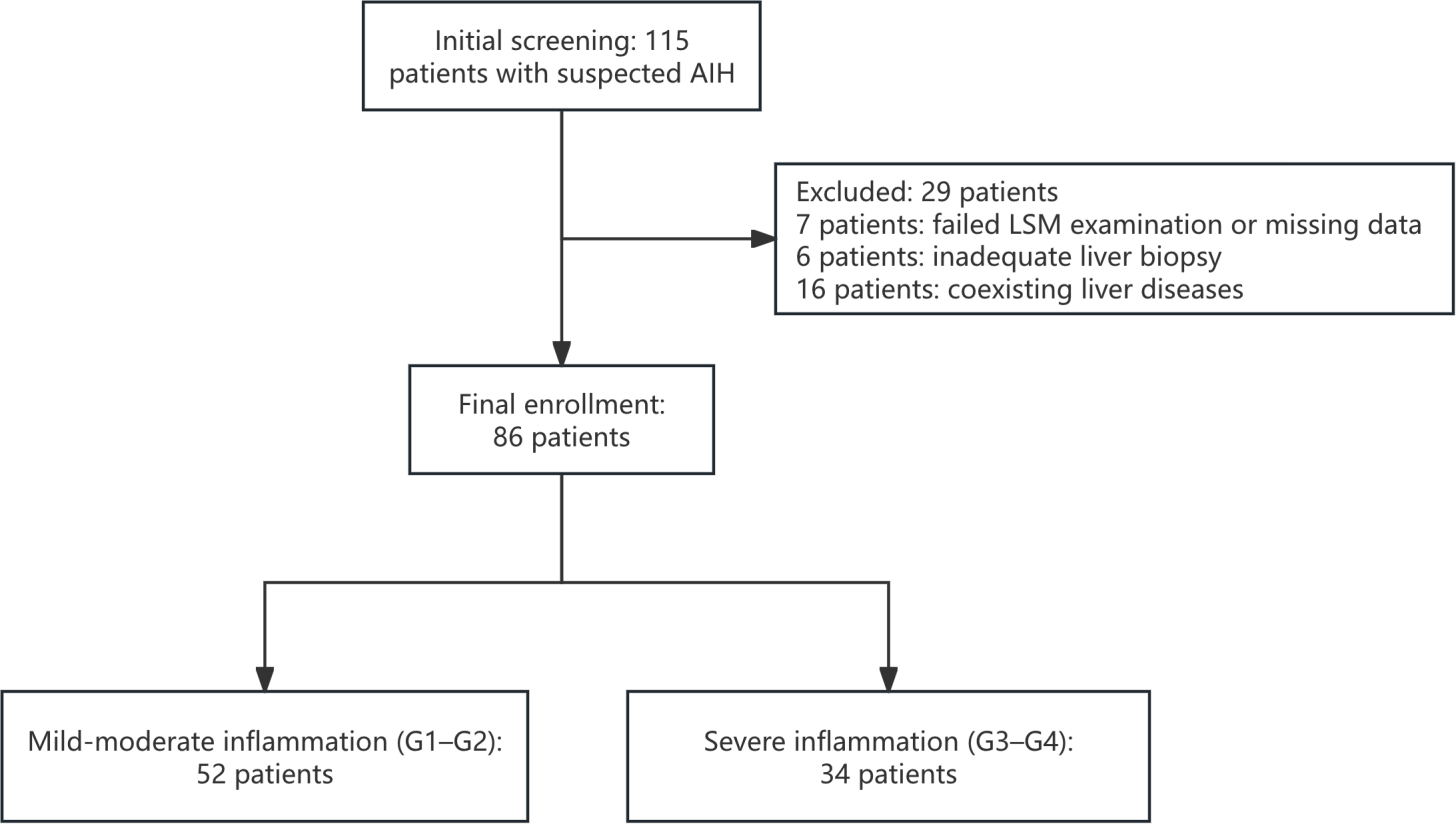
Flowchart of patient enrollment and exclusion in this retrospective cohort study.

A total of 115 patients with AIH were initially screened. 29 patients were excluded (7 patients: failed LSM examination or missing LSM data due to technical issues (e.g., high BMI, poor acoustic window); 6 patients: inadequate liver biopsy specimens; 16 patients: coexisting other chronic liver diseases). Finally, 86 AIH patients were enrolled (**Figure 1**). Among them, 79 (91.9%) were female and 7 (8.1%) were male. The mean age was (54.1 ± 9.1) years (range, 30–75 years). The mean BMI was (23.1 ± 3.5) kg/m², including 9 (10.5%) patients with BMI <18.5 kg/m², 48 (55.8%) with 18.5–23.9 kg/m², 24 (27.9%) with 24–27.9 kg/m², and 5 (5.8%) with ≥28 kg/m².

**Figure 2:**
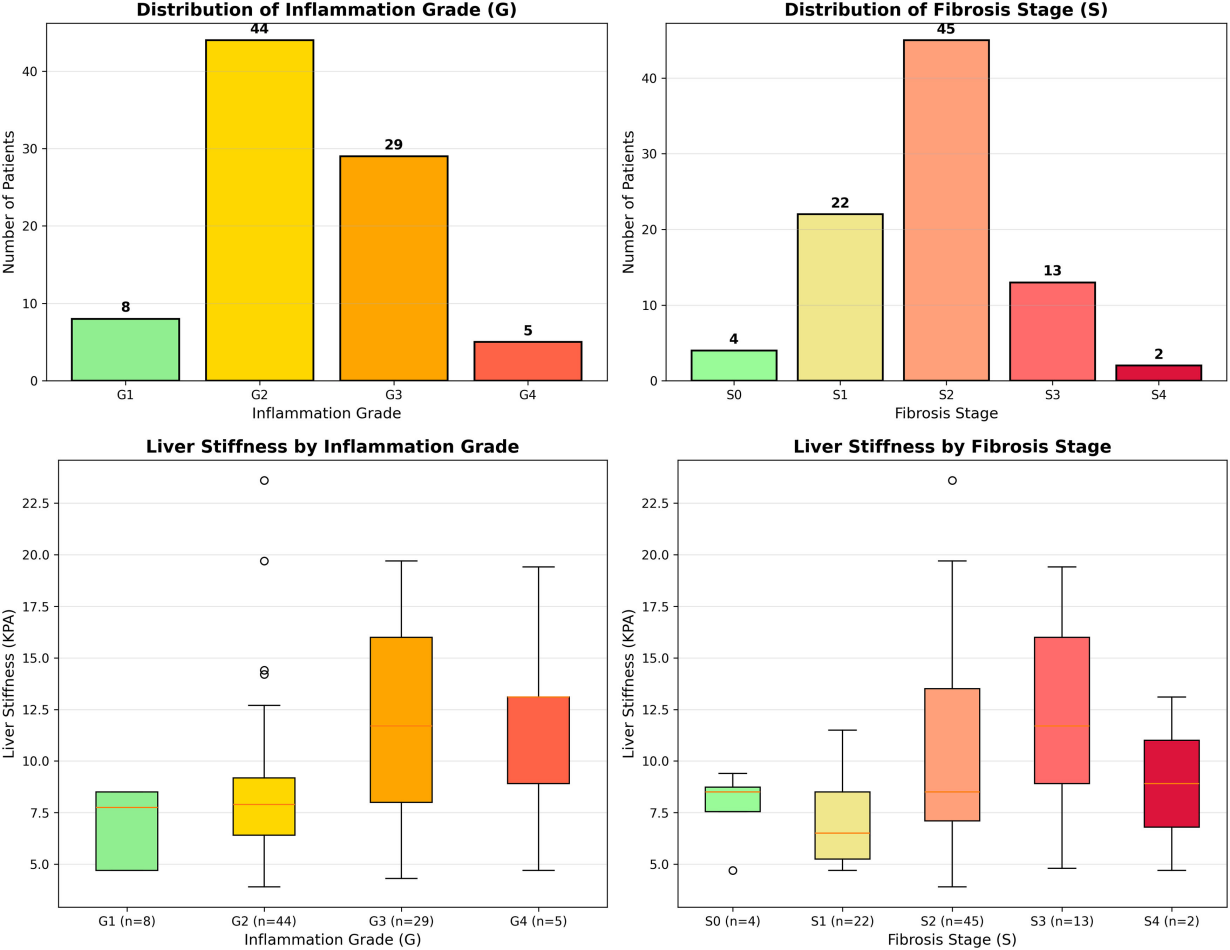
Liver stiffness measurement (LSM) values stratified by fibrosis stage and inflammatory activity. Liver stiffness measurement (LSM) values in AIH patients, stratified by fibrosis stage (S0–S1, S2, S3–S4) and inflammatory activity (G1–G2: mild-to-moderate inflammation; G3–G4: severe inflammation). Significant differences were observed between inflammatory groups in S0–S1 (P = 0.002) and S2 (P = 0.031), but not in S3–S4 (P = 0.208). LSM values in AIH patients with mild-to-moderate (G1–G2, blue) versus severe (G3–G4, red) inflammation, stratified by fibrosis stage (S0–S1, S2, S3–S4). Significant differences were found between groups in S0–S1 (P = 0.002) and S2 (P = 0.031), but not in S3–S4 (P = 0.208).

Patients were stratified by inflammatory grade: 52 in the mild-to-moderate inflammation group (G1-G2), including 8 (15.4%) with G1 and 44 (84.6%) with G2; and 34 in the severe inflammation group (G3-G4), including 29 (85.3%) with G3 and 5 (14.7%) with G4. No significant differences were observed between the two groups in age, sex, BMI, or treatment status (all P > 0.05), indicating good comparability (**Table 1**; **Figure 2**).

**Table 1:** Baseline characteristics of 86 patients with autoimmune hepatitis stratified by inflammatory activity.

Characteristic	Total (n=86)	G1–G2 (n=52)	G3–G4 (n=34)	P-value
Age, years, mean ± SD	54.1 ± 9.1	53.5 ± 8.2	55.0 ± 10.4	0.624
Female, n (%)	79 (91.9)	49 (94.2)	30 (88.2)	1.000
BMI, kg/m², mean ± SD	23.1 ± 3.5	23.4 ± 3.9	22.6 ± 2.9	0.668
BMI category, n (%)				0.811
<18.5	9 (10.5)	6 (11.5)	3 (8.8)	
18.5–23.9	48 (55.8)	29 (55.8)	19 (55.9)	
24.0–27.9	24 (27.9)	14 (26.9)	10 (29.4)	
≥28.0	5 (5.8)	3 (5.8)	2 (5.9)	
Liver stiffness, mean ± SD, kPa	9.6 ± 4.5	8.2 ± 3.6	11.7 ± 4.8	<0.001
Inflammatory grade, n (%)				<0.001
G1	8 (9.3)	8 (15.4)	0 (0.0)	
G2	44 (51.2)	44 (84.6)	0 (0.0)	
G3	29 (33.7)	0 (0.0)	29 (85.3)	
G4	5 (5.8)	0 (0.0)	5 (14.7)	
Fibrosis stage, n (%)				<0.001
S0	4 (4.7)	4 (7.7)	0 (0)	
S1	22 (25.6)	20 (38.5)	2 (5.9)	
S2	45 (52.3)	26 (50.0)	19 (55.9)	
S3	13 (15.1)	2 (3.8)	11(32.3)	
S4	2 (2.3)	0 (0)	2 (5.9)	

BMI, body mass index; G, inflammatory grade; S, fibrosis stage; SD, standard deviation.

Continuous variables are expressed as mean ± standard deviation and compared using independent-samples t-test.

Categorical variables are expressed as number (percentage) and compared using χ² test (Fisher exact test for expected frequency <5).

### Diagnostic efficacy of liver stiffness values in the overall population

**Figure 3:**
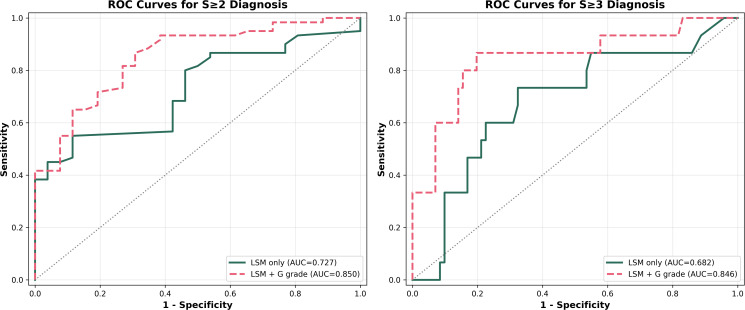
Receiver operating characteristic (ROC) curves of liver stiffness measurement (LSM) for diagnosing significant fibrosis (S≥2, S≥3) in overall AIH patients and subgroups stratified by inflammatory activity. The AUC, optimal cutoff value, and 95% confidence interval (CI) are presented for the total cohort, mild-to-moderate inflammation group (G1–G2), and severe inflammation group (G3–G4), respectively.

Among the 86 AIH patients, LSM ranged from 3.9 to 23.6 kPa, with a mean of (9.6 ± 4.5) kPa. ROC curve analysis showed that for the diagnosis of S≥2 fibrosis, the AUC of LSM was 0.73 (95% CI: 0.62–0.83), with an optimal cutoff of 8.2 kPa, corresponding sensitivity of 55.0%, specificity of 88.5%, PPV of 91.7%, and NPV of 46.3%. For the diagnosis of S≥3 fibrosis, the AUC was 0.68 (95% CI: 0.50–0.83), with an optimal cutoff of 8.9 kPa, sensitivity of 73.3%, and specificity of 67.6%. The diagnostic performance for S≥3 was suboptimal, indicating the need for improved diagnostic strategies (**Figure 3**).

### Diagnostic efficacy of liver stiffness values under inflammatory stratification

The AUC of LSM for diagnosing S≥2 fibrosis was 0.85 (95% CI: 0.76–0.94) in the G1–G2 group, which was significantly higher than 0.69 (95% CI: 0.54–0.84) in the G3–G4 group (DeLong test: Z = 2.13, P = 0.033).

The optimal cutoff was 7.8 kPa in the G1–G2 group, with sensitivity of 87.9% and specificity of 76.2%. The optimal cutoff was 9.1 kPa in the G3–G4 group, with sensitivity of 70.4% and specificity of 64.3%. Differences in sensitivity and specificity between the two groups were statistically significant (P<0.05) (**Table 2**).

**Table 2:** Diagnostic performance of LSM for significant fibrosis (S≥2) stratified by inflammatory activity.

Variable	G1–G2 (n=52)	G3–G4 (n=34)	P-value
Cut-off value, kPa	7.8	9.1	–
AUC (95% CI)	0.85 (0.76–0.94)	0.69 (0.54–0.84)	0.033
Sensitivity, %	87.9	70.4	<0.05
Specificity, %	76.2	64.3	<0.05
PPV, %	82.6	73.1	–
NPV, %	82.1	61.4	–

### Calibration model and validation of hardness values for the G3–G4 group

To address the reduced diagnostic performance in the severe inflammation group (G3–G4), multivariate Logistic regression analysis was performed with S≥2 fibrosis as the dependent variable and raw LSM, inflammatory grade (G), and their interaction term (LSM × G) as independent variables. Multivariate logistic regression revealed a significant interaction between LSM and inflammatory grade (β=0.46, 95% CI: 0.18–0.74, P = 0.008). A correction formula was accordingly established:

### Corrected LSM = raw LSM − 0.4 × inflammatory grade (subtract 1.2 kPa for G3, 1.6 kPa for G4)

The correction coefficient (0.4) was derived from multivariate logistic regression (**Supplementary Table 1**). After correction for S≥2 diagnosis, the calibration model achieved an AUC of 0.85 (95% CI: 0.76–0.93), significantly higher than raw LSM alone (AUC = 0.73, DeLong test: Z = 2.89, P = 0.004). Sensitivity improved from 55.0% to 86.7%, and specificity was 69.2%. For S≥3 diagnosis, the AUC was 0.85 (95% CI: 0.72–0.96), significantly higher than raw LSM (AUC = 0.68, DeLong test: Z = 3.12, P = 0.002). Sensitivity was 86.7% and specificity was 80.3% (**Table 3**; **Figure 3**).

**Table 3:** Comparison of diagnostic performance for before and after LSM correction in the group.

Variable	Before correction	After correction	P-value
S≥2			
AUC (95% CI)	0.73 (0.62–0.83)	0.85 (0.76–0.93)	0.004
Sensitivity, %	55	86.7	<0.001
Specificity, %	88.5	69.2	<0.05
PPV, %	91.7	86.7	0.412
NPV, %	46.3	75	0.012
S≥3			
AUC (95% CI)	0.68 (0.50–0.83)	0.85 (0.72–0.96)	0.002
Sensitivity, %	73.3	86.7	0.317
Specificity, %	67.6	80.3	0.089
PPV, %	32.4	48.1	0.156
NPV, %	92.3	96.6	0.243

### Exploratory analysis in the G1–G2 subgroup

To explore whether the correction formula was applicable to patients with mild-to-moderate inflammation, we applied the same formula to the G1–G2 subgroup. After correction, the AUC for diagnosing S≥2 fibrosis changed from 0.85 to 0.84 (95% CI: 0.75–0.93; P = 0.682). Sensitivity and specificity also showed no significant improvement (P > 0.05). These findings indicated that the inflammation-based correction was not necessary for the G1–G2 subgroup.

**Figure 4:**
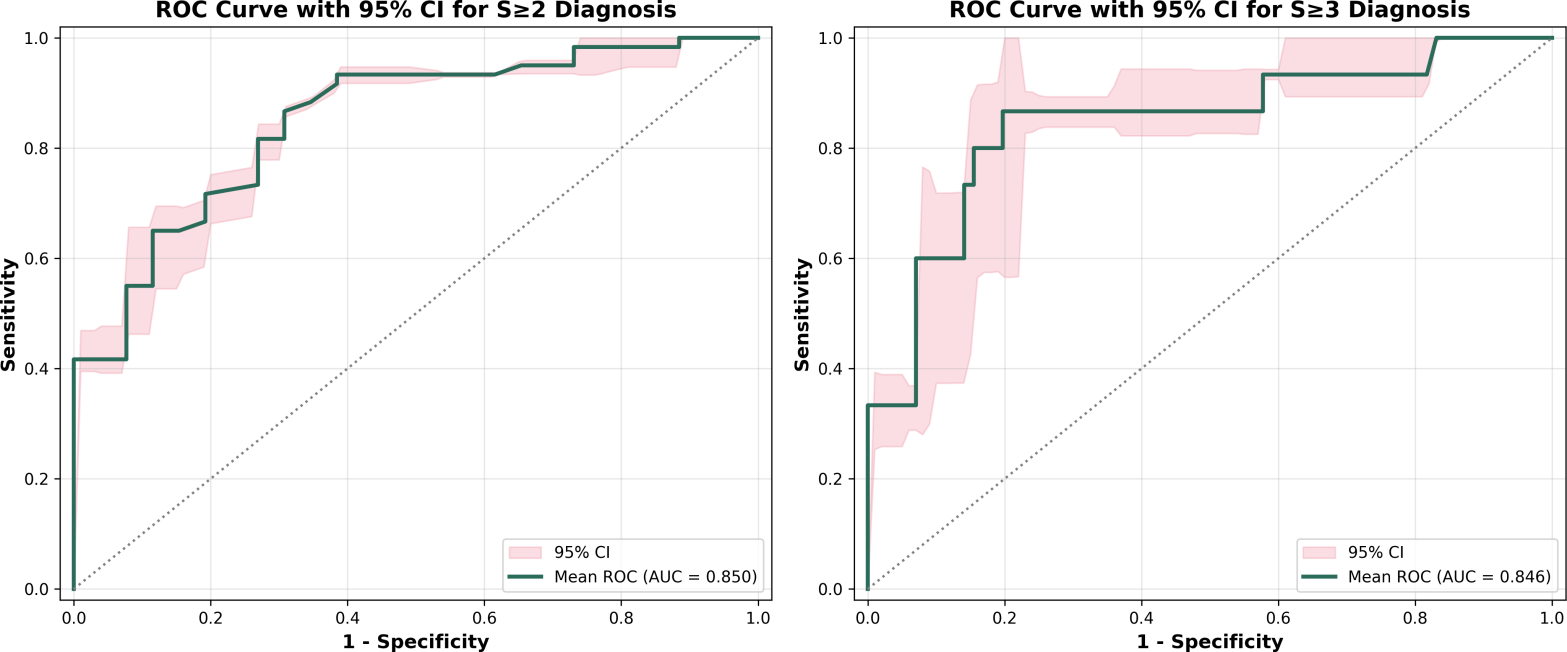
Comparison of ROC curves for diagnosing significant fibrosis (S≥2, S≥3) using raw LSM and inflammation-corrected LSM in the severe inflammation subgroup (G3–G4). Receiver operating characteristic (ROC) curves for diagnosing significant fibrosis (S≥2) using raw LSM and inflammation-corrected LSM in the severe inflammation subgroup (G3–G4). Correction formula: corrected LSM = raw LSM − 0.4 × inflammatory grade. After correction, the AUC increased from 0.69 to 0.85 (95% CI: 0.76–0.93) (P<0.05, DeLong test).

**Figure 5:**
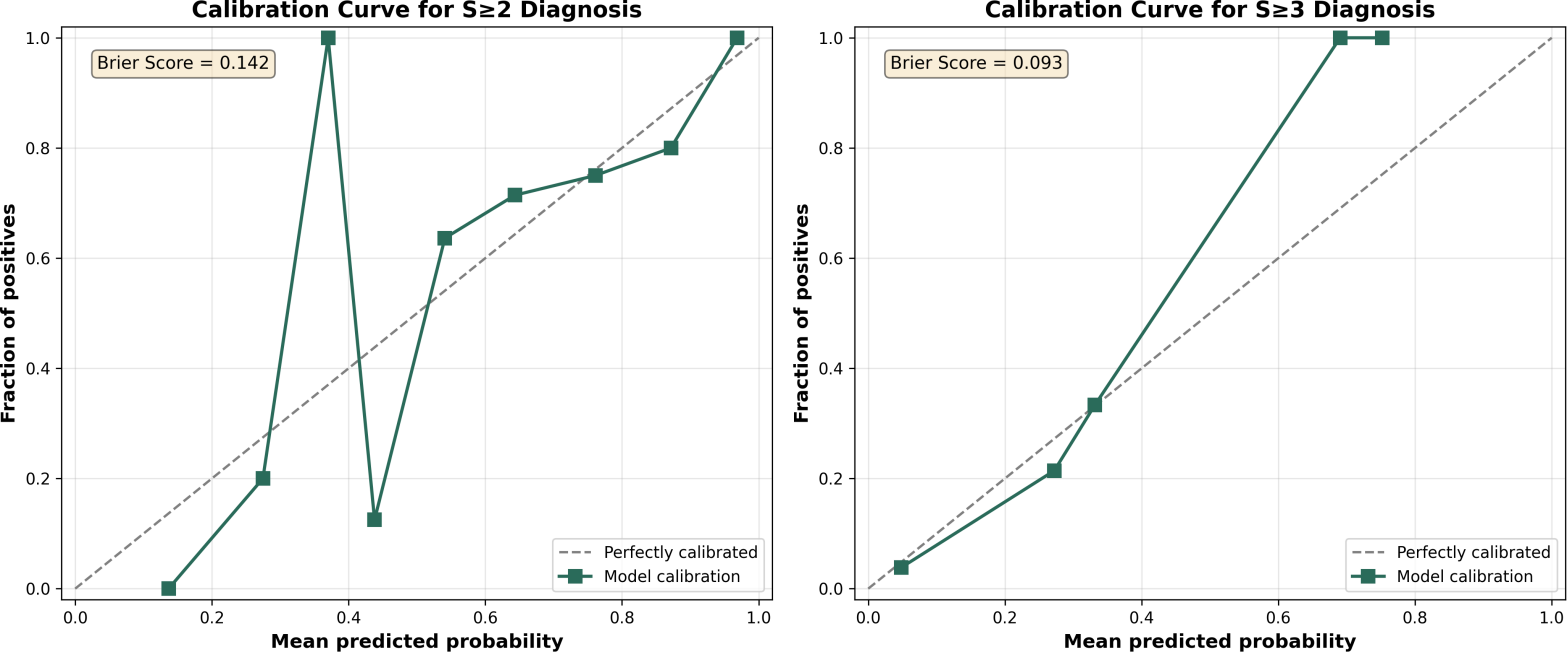
Bootstrap calibration curve of the inflammation-corrected LSM model for predicting significant fibrosis. Bootstrap calibration curve of the inflammation-corrected LSM model for predicting significant fibrosis (S≥2, S≥3) based on 1000 bootstrap resamples. The diagonal line represents perfect prediction; the solid line shows the predictive performance of the model, indicating minimal overfitting and good stability.

Internal validation using the Bootstrap method (1000 resamples) showed that the AUC for S≥2 diagnosis was 0.85 (95% CI: 0.83–0.85), and for S≥3 diagnosis was 0.84 (95% CI: 0.83–0.85), which were highly consistent with the original estimates, confirming good model stability. The Brier scores were 0.146 for S≥2 and 0.098 for S≥3, indicating accurate probability predictions (**Figures 4**, **5**). The proposed clinical workflow for interpreting LSM in AIH patients using the inflammation-based correction strategy is shown in **Supplementary Figure 1**.

## Discussion

Although LSM has been increasingly used for non-invasive fibrosis assessment in chronic liver diseases, the specific impact of inflammatory activity on its diagnostic performance in autoimmune hepatitis remains unclear (15). This study demonstrates that severe inflammation significantly compromises diagnostic accuracy, with the area under the curve falling from 0.85 in the mild-to-moderate inflammation group to 0.69 in the severe inflammation group. More importantly, we developed a simple inflammation-based correction model that restores the area under the curve to 0.85 in severely inflamed patients, achieving diagnostic performance comparable to that observed in patients with lower inflammatory activity. This model can effectively restore the diagnostic capability for patients with severe inflammation, providing a practical tool for assessing liver fibrosis in this special population.

The overall diagnostic performance of LSM in our cohort (AUC = 0.73) was consistent with previous systematic reviews and prospective studies in AIH, falling within the range of moderate to good diagnostic accuracy. Previous meta-analyses have reported pooled AUC values of approximately 0.88, 0.88, and 0.92 for VCTE in detecting significant fibrosis, advanced fibrosis, and cirrhosis in AIH, respectively (11). In addition, current guidelines indicate that the diagnostic accuracy of VCTE is significantly better 6–12 months after treatment than within the first 3 months (AUC 0.97–1.0 vs. 0.68–0.80), suggesting that LSM more accurately reflects fibrosis stage once inflammation has subsided (15). Thus, our results may be considered a robust representation of LSM performance under real-world conditions, where inflammatory activity is a confounding factor.

In meta-analyses of chronic hepatitis B, the pooled AUC values of LSM for significant fibrosis and cirrhosis were 0.82 and 0.91, respectively (16). It is well documented that in chronic viral hepatitis, LSM is driven not only by fibrosis but also by intrahepatic inflammation. During acute flares or when ALT levels are elevated, LSM can increase to values in the “cirrhosis range”. Moreover, the early decline in LSM after treatment mainly reflects reduced inflammation rather than fibrosis regression (17). Current guidelines also emphasize that the confounding effect of inflammation on LSM in chronic viral hepatitis has been widely reported, and thus caution is needed when comparing the diagnostic performance of LSM across different etiologies. Our stratification analysis directly supports this interpretation: after excluding patients with severe inflammation, the diagnostic performance in the mild-to-moderate inflammation group (AUC 0.85) approached the level seen in viral hepatitis, whereas the severe inflammation group showed substantially poorer performance (AUC 0.69). Our study extends this observation by demonstrating that the optimal diagnostic threshold is substantially higher in severely inflamed patients compared to those with mild-to-moderate inflammation. This implies that applying a single threshold to all patients would systematically overestimate fibrosis in those with active inflammation.

The observed inflation of LSM in severely inflamed AIH patients can be explained by several mechanisms. The interface hepatitis that defines active AIH is characterized by dense infiltration of plasma cells and lymphocytes into the periportal region. This cellular infiltration directly increases local tissue density and elastic modulus, an effect that is more pronounced in AIH than in viral hepatitis due to the predominance of plasma cells (18). In patients with active AIH, hepatic inflammation can increase LSM values through reversible mechanisms, leading to falsely elevated LSM readings in those with severe inflammation. This phenomenon is primarily driven by the direct effects of inflammatory activity on liver tissue, rather than by true fibrotic progression. As a result, LSM may overestimate the degree of fibrosis in severely inflamed patients, making accurate staging challenging (19, 20). Our findings highlight this limitation and support the use of an inflammation-based correction model to improve the accuracy of fibrosis assessment in AIH.

Our findings offer a practical framework for interpreting liver stiffness measurement in AIH. For initial screening, a threshold of 8.2 kPa can be applied. However, when patients present with clinical clues of severe inflammation—such as markedly elevated aminotransferases or histologically confirmed G3-G4 grade—the raw stiffness value should not be interpreted directly (21, 22). In these patients, we recommend calculating the corrected liver stiffness measurement using our formula. A corrected value above 8.2 kPa suggests true significant fibrosis, suggesting intensified therapy or repeat biopsy. A corrected value below 8.2 kPa indicates that the elevated stiffness is primarily attributable to inflammation rather than fibrosis, avoiding unnecessary invasive procedures. Furthermore, exploratory analysis confirmed that correction was unnecessary in the G1–G2 subgroup, as it did not improve diagnostic performance, supporting the targeted use of this formula only in severe inflammation. Notably, the correction model performed similarly in untreated patients, supporting its applicability across different treatment stages. Furthermore, we present a clinical decision workflow to enable quick and straightforward judgment.

A critical paradox of the present model is its dependence on histological inflammatory grade (G), which requires invasive liver biopsy. This limits the real-world clinical utility of the correction formula, as many AIH patients are managed without immediate histological evaluation. In clinical scenarios where liver biopsy is unavailable or delayed, serum ALT and AST levels may serve as a surrogate marker for inflammatory activity. Markedly elevated transaminases (>5× ULN) suggest severe inflammation, and the higher LSM cutoff (9.1 kPa) or corrected formula may be applied. However, histologic inflammatory grade remains the gold standard, and biopsy is still recommended when feasible for accurate evaluation (23). A grey zone between 7.8 kPa and 9.1 kPa exists, in which LSM values are strongly affected by hepatic inflammation. For a patient with an LSM value of 8.5 kPa and unknown inflammatory status, clinicians should first evaluate ALT/AST levels to estimate inflammatory activity. If enzymes are markedly elevated, the LSM value is likely inflated by inflammation; if normal, the value more likely indicates significant fibrosis. Serial LSM measurements after anti-inflammatory treatment are recommended for further clarification (24). Future studies are warranted to develop a fully non-invasive model incorporating biochemical inflammatory markers, rather than histological grade, to resolve this paradox and broaden the clinical applicability of inflammation-adjusted LSM interpretation.

This study has several strengths. It represents one of the largest single-center cohorts of pure AIH patients (n=86) specifically examining liver stiffness measurement for fibrosis assessment, with rigorous exclusion of other liver diseases that could confound the analysis. The proposed correction formula is simple, requiring only the inflammatory grade, and can be easily implemented in clinical practice. Several limitations should be acknowledged. First, the single-center retrospective design may introduce selection bias. Specifically, patients undergoing biopsy may have had higher pretest probability of significant fibrosis, which could modestly inflate diagnostic performance estimates. Although the present study met the required sample size determined by *a priori* power analysis, the cohort size (n=86) remains relatively limited for developing a predictive model. To minimize overfitting, we performed 1000-resample bootstrap validation, which yielded a low optimism (0.02) and stable optimism-corrected AUC (0.85), supporting that the model was well-calibrated and that overfitting was well-controlled. However, our primary finding—that inflammation reduces diagnostic accuracy—is a comparative measure that is less susceptible to this bias. Second, although we performed bootstrap internal validation, external validation in an independent multi-center cohort is needed to confirm the generalizability of our correction model; we are currently planning such a validation study. Third, due to the limitation of our sample size, we did not perform subgroup analyses according to different subtypes of AIH. Fourth, while FibroTouch and FibroScan show high concordance (ρ=0.87) (25), the specific thresholds proposed here may require calibration for other devices. Finally, we only compare the calibration model with mature serum biomarkers such as FIB-4 or APRI, but we have not explored its deep-seated significance (**Supplementary Table 2**); future studies should explore whether combining liver stiffness measurement with these models provides additional diagnostic value.

## Conclusion

In conclusion, the diagnostic performance of LSM is significantly impaired by severe inflammatory activity in AIH, leading to pseudo-elevation of liver stiffness. Our inflammation−stratified correction model effectively improves diagnostic accuracy in patients with high−grade inflammation, providing a practical noninvasive strategy for individualized fibrosis assessment in AIH. This approach warrants further validation before routine clinical use.

## Data Availability

The original contributions presented in the study are included in the article/**Supplementary Material**. Further inquiries can be directed to the corresponding authors.
