# Supplementary material for: Inflammatory activity stratification improves liver stiffness diagnosis of fibrosis in autoimmune hepatitis
Source: Front Immunol. 2026 Jun 10;17:1865212. doi: 10.3389/fimmu.2026.1865212 (PMC13291146; doi:10.3389/fimmu.2026.1865212)
Supplement: Supplementary file 1 [file SupplementaryFile1.docx]

**Supplementary Material**

**Supplementary Table 1.** Multivariate Logistic Regression Model for Significant Fibrosis (S ≥ 2)

| **Variable** | **Coefficient** | **Standard Error** | **Odds Ratio** | **95% CI** | **P-value** |
| --- | --- | --- | --- | --- | --- |
|  |  |  |  |  |  |
| Age (years) | 0.021 | 0.032 | 1.021 | 0.959–1.088 | 0.512 |
| BMI (kg/m²) | 0.035 | 0.044 | 1.036 | 0.950–1.129 | 0.407 |
| Liver stiffness measurement (LSM, kPa) | 0.326 | 0.091 | 1.385 | 1.159–1.656 | <0.001 |
| Inflammatory grade (G) | 0.4 | 0.167 | 1.492 | 1.075–2.071 | 0.016 |
| LSM × Inflammatory grade interaction | 0.460 | 0.143 | 1.584 | 1.189–2.113 | 0.008 |
| Constant | −4.216 | 1.253 | — | — | <0.001 |

Supplementary Note

Multivariate logistic regression was performed to identify independent factors associated with significant fibrosis (S ≥ 2).

Liver stiffness measurement (LSM), histological inflammatory grade (G), and their interaction term (LSM × G) were confirmed as independent predictive factors.

The interaction term coefficient (β = 0.46) and inflammatory grade coefficient (β = 0.40) were combined to establish the inflammation‑corrected LSM formula used in the main text:

Corrected LSM = Raw LSM − 0.4 × Inflammatory grade

This formula was derived from the multivariate regression model and consistent with the main text analysis.

## **Supplementary Table 2.** Diagnostic performance of non‑invasive markers for predicting significant fibrosis (S ≥ 2) in AIH patients

| **Marker** | **AUC (95% CI)** | **Sensitivity (%)** | **Specificity (%)** |
| --- | --- | --- | --- |
| **Calibration Model LSM** | 0.85 (0.76–0.93) | 86.7 | 69.2 |
| **Raw LSM** | 0.73 (0.62–0.83) | 55.0 | 88.5 |
| **APRI** | 0.71 (0.59–0.80) | 68.5 | 66.3 |
| **FIB-4** | 0.72 (0.61–0.82) | 70.1 | 68.4 |

Note: Abbreviation: LSM, liver stiffness measurement; AUC, area under the receiver operating characteristic curve; CI, confidence interval.

This table compares the diagnostic performance of raw liver stiffness measurement (LSM), APRI, FIB-4, and Calibration Model LSM for significant fibrosis (S ≥ 2) in patients with autoimmune hepatitis, demonstrating the improved accuracy after inflammation stratification and correction.

**Supplementary Figure 1. Proposed clinical workflow for interpreting LSM in AIH patients using the inflammation-based correction strategy.**

**
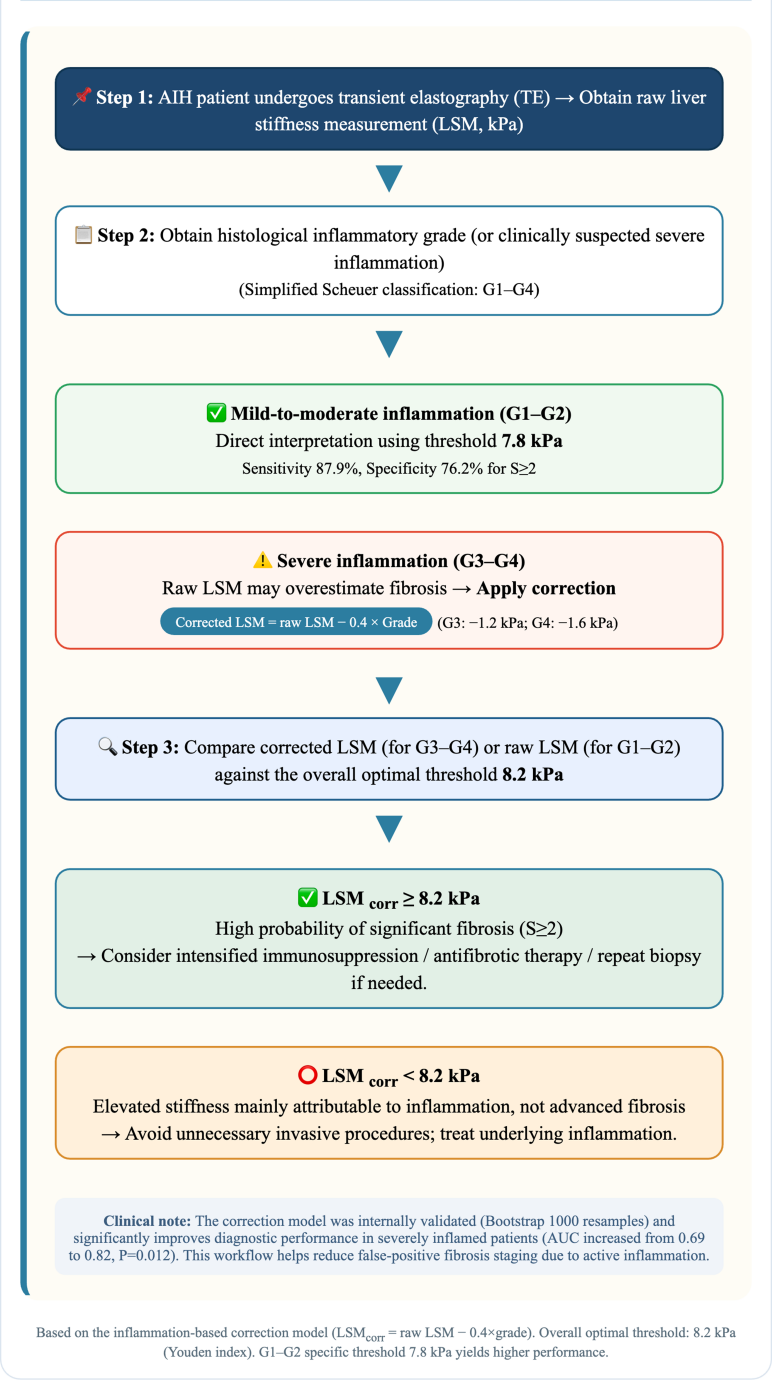
**

The flowchart illustrates LSM testing, inflammatory grading, application of the correction formula in G3–G4 patients, and clinical decision-making for significant fibrosis.
